# Gliclazide in Binary and Ternary Systems Improves Physicochemical Properties, Bioactivity, and Antioxidant Activity

**DOI:** 10.1155/2022/2100092

**Published:** 2022-11-25

**Authors:** Muhammad Ibrahim, Shehla Munir, Sarfraz Ahmed, Adeel Hussain Chughtai, Waqas Ahmad, Jallat Khan, Mogana Das Murtey, Hira Ijaz, Suvash Chandra Ojha

**Affiliations:** ^1^Department of Biochemistry, Bahauddin Zakariya University, Multan 60800, Pakistan; ^2^Department of Basic Sciences, University of Veterinary and Animal Sciences Lahore, Narowal Campus, Narowal 51600, Pakistan; ^3^Institute of Chemical Sciences, Bahauddin Zakariya University, Multan 60800, Pakistan; ^4^Department of Clinical Sciences, University of Veterinary and Animal Sciences Lahore, Narowal Campus, Narowal 51600, Pakistan; ^5^Department of Chemistry, Khwaja Fareed University of Engineering & Information Technology, Rahim Yar Khan 64200, Pakistan; ^6^Basic Sciences and Oral Biology Unit, School of Dental Sciences, Health Campus, Universiti Sains Malaysia, Kubang Kerian 16150, Kelantan, Malaysia; ^7^Department of Pharmacy, Pak–Austria Fachhochschule Institute of Applied Sciences and Technology, Mang, Haripur 22620, Khyber Pakhtunkhwa, Pakistan; ^8^Department of Infectious Diseases, The Affiliated Hospital of Southwest Medical University, Luzhou 646000, China; ^9^Southwest Medical University, Jiangyang District, Luzhou 646000 Sichuan, China

## Abstract

The poor solubility of the antidiabetic drug gliclazide (Glc) is due to its hydrophobic nature. This research is aimed at improving Glc's solubility and drug release profile, as well as at investigating additional benefits such as bioactivity and antioxidant activity, by forming binary complexes with HP*β*CD at different *w*/*w* ratios (1 : 1, 1 : 2.5, 1 : 4, and 1 : 9) and ternary complexes with HP*β*CD and Tryp at 1 : 1 : 1, 1 : 1 : 0.27, 1 : 2.5 : 0.27, 1 : 3.6 : 3.6, 1 : 4 : 1, and 1 : 9 : 1, respectively. Complexes were prepared by the physical mixing (PM) and solvent evaporation (SE) methods. The prepared inclusion complexes were meticulously characterized by X-ray diffractometry (XRD), scanning electron microscopy (SEM), and attenuated total reflectance-Fourier transform infrared (ATR-FTIR) spectra. To verify our findings, the inclusion complexes were evaluated by equilibrium solubility, *in vitro* drug release profile, kinetic models, and antidiabetic and antioxidant activities in animal models. Our results demonstrated that the solubility and drug release profile were found to be enhanced through binary as well as ternary complexes. Notably, ternary complexes with a ratio of 1 : 9 : 1 showed the highest solubility and drug release profile compared to all other preparations. Data on antioxidant activity indicated that the ternary complex had the higher total antioxidant status (TAS), superoxide dismutase (SOD), and catalase (CAT) activity than the binary complex and Glc alone, in contrast to the diabetic group. *In vivo* antidiabetic activity data revealed a high percentage reduction in the blood glucose level by ternary complexes (49–52%) compared to the binary complexes (45–46%; *p* ≤ 0.05). HP*β*CD and Tryp provide a new platform for overcoming the challenges associated with poorly soluble Glc by providing greater complexing and solubilizing capabilities and imparting ancillary benefits to improve the drug's antidiabetic and antioxidant activities.

## 1. Introduction

**Figure 1:**
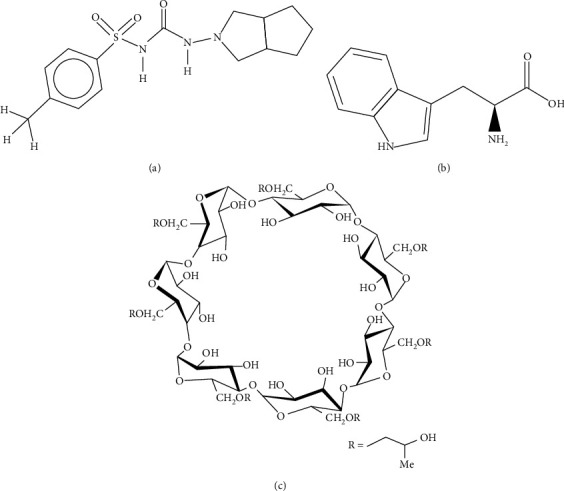
Structure of (a) Glc, (b) Tryp, and (c) HP*β*CD.

Diabetes mellitus is a metabolic disorder characterized by high-blood glucose levels caused by deficiencies in insulin secretion or insulin action resistance. Scientists theorize that this disorder amplifies oxidative stress levels due to depletion in ascorbic acid [1]. Glc (1-[(4-methylbenzene) sulfonyl]-3-[octahydrocyclopenta [c] pyrrol-2-yl] urea) is a derivative of sulfonylurea (Figure 1(a)) [2], a BCS class II drug, which has low solubility and high permeability and exhibits dissolution rate-limited absorption. According to the Burnham Center for Chemical Genomics, Glc solubility in water is 42.6 g/mL, which is used to treat type II diabetes mellitus, reduces hepatic gluconeogenesis, increases fibrinolysis, and inhibits platelet accumulation [3]. Glc has the chemical formula C_15_H_21_N_3_O_3_S, a molecular weight of 323.42 g/mol, a log *p* value of 2.6, and a pKa of 5.8 [4]. Glc exists as a white crystalline powder that is odorless and tasteless and has a melting point of 165–170°C, ensuring stability under normal conditions [5].

Glc has recently received considerable attention for revealing other pharmacological properties like antioxidant and anti-inflammatory activities *in vitro* and in animal models. When compared to other sulfonylurea drugs, Glc has a higher potential for free radical scavenging because the scavenging part of the molecule is an aminoazabicyclo-octane ring, which is not evident in other sulfonylurea drugs [6]. According to research findings [7], peak plasma levels of Glccan be reached in 2–8 hours. The time to attain peak plasma concentrations (tmax) has been reported to vary greatly between and within individuals, which is attributed to dissemination in the extracellular fluid, liver, lungs, skin, kidney, cardiac, and skeletal muscles. Age-related variations in plasma peak concentrations (Cmax) have been reported and oral administration of 40–120 mg of Glc results in Cmax values ranging from 2.2 to 8.0 *μ*g/mL within 2–8 hours. Regular Glc administration raises Cmax and tmax levels, and after 48 hours of 40–120 mg doses, a steady-state concentration is achieved. Because of its high affinity for protein binding (85–97%), a small volume of Glc (13 to 24 L) is distributed in both healthy volunteers and patients. Glc half-life (t1/2) is approximately 8.1 to 20.5 hrs, and plasma clearance is 13 mL/min (0.78 L/hr). Because the drug is easily metabolized and excreted in the urine, renal insufficiencies have no effect on Glc's pharmacokinetic properties [7].

Glc's poor solubility due to its hydrophobic nature is a major flaw in formulation, making it challenging for scientists to produce dosage forms that may improve its solubility and bioavailability [8]. Poor solubility and extensive first-pass effect are credited for wide intraindividual variation in its bioavailability, which has a primary influence on the pharmacokinetics of the drug. Furthermore, drug dissolution is a rate-limiting step in drug absorption, which adds extra to the challenges. To improve drug bioactivity, solubility and dissolution in gastrointestinal fluids are considered vital counterparts [9]. At present, Glc solubility can be improved utilizing a variety of physical and chemical approaches such as solid dispersion, cogrinding, *in situ* micronization, nanospheres, solid complexes, and prodrug formation [10]. Recent studies claimed that cocrystallization [11, 12] could improve Glc's solubility, release profile, and other physicochemical properties by forming its solid dispersion with plasdone K-90, plasdone K-30, and eudragit RS-100 [13, 14] using hydrophilic fumed silica particles [15]. In addition, previous studies investigated Glc's binary and ternary complexes using different polymers, including polyethylene glycol 6000, pluronic F68, poloxamer 188, hydroxypropyl methylcellulose, and eudragit polymers. These polymer-based complexes significantly increased the dissolution rate and total absorption of the drug from the small intestine [16, 17].

New technologies and raw materials assisted in developing stable, safe, and efficient drug carriers with fascinating strategies to develop updated formulations, which improve efficacy, solubility, and bioavailability [18]. HP*β*CD is demonstrated as a type of cyclodextrin with seven glucose units and hydroxypropyl as a substituent (Figure 1(c)). Previous research indicates that it has satisfactory inclusion ability, improves water solubility, and is less toxic than *β*-cyclodextrin. HP*β*CD is produced by the replacement of the hydroxyl group by the hydroxypropyl substituent at 2, 3, and 6 positions [19]. According to the product description by Sigma-Aldrich, it is a white solid with the molecular formula (C_6_H_9_O_5_)_7_(C_3_H_7_O)_45_, an average molecular weight of 1396 (anhydrated), and a substitute of 0.67 hydroxypropyl groups per glucose unit, highly soluble in water 45 g/100 mL. The literature shows that cyclodextrin complexes boost and maintain the antioxidant activity of drugs, providing an additional benefit [20].

Recent progress in pharmaceutics has captivated scientists' attention toward improving drug solubility, stability, and dissolution using amino acids as coamorphous systems. The homogenous mixture forms intermolecular interaction, thereby increasing glass transition temperature (Tg), which enhances solubility and stability of the drug with the least excipient usage. Amino acid interacts with drugs via H-bond, *π*–*π*, and ionic interaction, which prevents drugs from becoming crystallized [8]. Tryptophan (Tryp) exhibits a strong flair or potential for stabilizing poorly water-soluble drugs [21] as it holds an indole group as a side chain of hydrogen that can be used in hydrogen bonding with drug molecule (Figure 1(b)).

Overall, Tryp is comprehensively presumed to be nonpolar. As an essential amino acid, it contributes to the synthesis of key metabolic products such as protein, kynurenine, serotonin, tryptamine, melatonin, coenzymes, and niacin [22]. The reactivity of tryptophan with highly reactive free radicals is attributed to its strong antioxidant properties. Its metabolites exhibited potential antioxidant activity.

To the best of our knowledge, there have been few studies on the ternary complexes of Glc for improving its solubility and release patterns. Hence, the present study is aimed at preparing the binary and ternary complexes of Glc with HP*β*CD and Tryp to improve its solubility and release patterns using a series of different *w*/*w* ratios via physical mixing (PM) and solvent evaporation (SE), followed by advanced experimental analyses. The bioactivity of complexes was deduced in animal models to validate our findings. Furthermore, the antioxidant activity of complexes was also investigated.

## 2. Material and Methods

### 2.1. Chemicals

The chemicals used in this study comprise Glc (Shandong Keyuan Pharmaceutical Co., China), methanol (Sigma-Aldrich, Germany), hydroxypropyl-*β*-cyclodextrin (Sigma-Aldrich, Germany), DL-tryptophan (BDH Chemicals, Poole, England), potassium dihydrogen phosphate (Duksan Pure Chemicals, Korea), sodium hydroxide (Duksan Pure Chemicals, Korea), magnesium stearate (Sigma-Aldrich, Germany), Starch (Fluka, USA), polyvinylpyrrolidone K 25 (Fluka, Germany), silica gel beads (Sigma-Aldrich, Germany), and alloxan monohydrate (Chem-Impex Int'l Inc., USA). All the materials used in the present study were of analytical grade.

### 2.2. Complexes Preparation

#### 2.2.1. Formation of Binary and Ternary Complexes by the Physical Mixing Method

The physical mixtures were prepared using two components (Glc : HP*β*CD) in binary systems in *w*/*w* ratios of 1 : 1, 1 : 2.5, 1 : 4, and 1 : 9 and three components (Glc: HP*β*CD : Tryp) in ternary systems in *w*/*w* ratios of 1 : 1 : 0.27, 1 : 1 : 1, 1 : 2.5 : 0.27, 1 : 3.6 : 3.6, 1 : 4 : 1, and 1 : 9 : 1, respectively. These ingredients were thoroughly ground, sieved (with a US 180 *μ*m sieve), and stored in dried brown bottles. These samples were stored at 25°C in a desiccator [23].

#### 2.2.2. Formation of Binary and Ternary Complexes through a Solvent Evaporation Method

Glc is insoluble in water and was dissolved in methanol (1 g/10 mL). HP*β*CD was solubilized in deionized water (45% *w*/*v*), and Tryp dissolution was performed in aqueous methanol (70%). For the preparation of a 1 molar solution, 204 g of tryptophan was solubilized in 700 mL of methanol and 300 mL of distilled water. All the components were accurately and validly weighed in accordance with the previously stated ratios, and complexes were prepared using the conventional solvent evaporation method [23].

#### 2.2.3. Characterization

*(1) Infrared Spectroscopic Studies*. Attenuated total reflection Fourier transform infrared (ATR-FTIR) spectra of pure Glc, HP*β*CD, Tryp, and all samples were obtained using an ATR-FTIR spectrometer (Brucker, Optics GmbH, Billerica, MA, USA) equipped with a mercury-cadmium-telluride detector. All samples were scanned at a resolution of 4 cm^−1^ over the wavenumber range of 400–4500 cm^−1^. Each spectrum was collected from 50 scans in the absorbance mode. Triplicate measurements were recorded, and mean values were used to ensure precision. In order to obtain FTIR spectra, the percent transmittance was plotted (*y*-axis) as a function of the wavenumber (*x*-axis). The spectral illustration was created using OriginPro 7.5 software.

*(2) Surface Morphological Study*. The samples prepared by PM and SE methods with the ratios of 1 : 1, 1 : 4, 1 : 9, 1 : 1 : 1, 1 : 2.5 : 0.27, 1 : 3.6 : 3.6, and 1 : 9 : 1 were designated for surface morphology study. The surface morphology, shape, nature, and height of pure Glc, HP*β*CD, Tryp, and the selected complexes were investigated through a scanning electron microscope (Carl Zeiss Microscopy Ltd.). The sample was fixed on a brass stub before being coated with a thin layer of gold to ensure good electrical conductivity while performing microscopic scans. Images were captured at magnification powers of 200, 250, 500, 1000, 1500, and 2500 *μ*m, with an accelerating voltage of 15 kV.

*(3) X-Ray Diffraction Studies*. X-ray diffractograms of pure Glc, HPCD, Tryp, and samples (1 : 1, 1 : 4, 1 : 9, 1 : 1 : 1, 1 : 2.5 : 0.27, 1 : 3.6 : 3.6, and 1 : 9 : 1) prepared by PM and SE were obtained using an X-ray diffractometer (Rigaku Corporation, Japan) with a HyPix-400 MF 2D hybrid pixel array detector at a voltage of 40 kV and a current of 30 mA. Cu was used as the targeting material. A modified system of diverging, receiving, and scattering slits of 1°, 0.2 mm, and 1° was utilized. The step angle was 0.05°, and the count time was 1 sec. The resulting patterns were recorded over a scan range of 5° to 50° at 2*θ*. OriginPro 7.5 software was used to create the comparison graphs.

#### 2.2.4. Equilibrium Solubility Studies

To determine solubility, 0.4 g of each sample containing binary and ternary complexes was added to 10 mL of deionized water. These mixtures were mixed thoroughly by a vortex mixer for 3–5 minutes and then kept on an orbital shaker at 150 rpm and 37°C for seven days to attain equilibrium. The samples were filtered through an AcrodiscGF filter syringe of 0.45 *μ*m (Pall Life Sciences), and 1 mL of each solution was diluted to 10 mL with deionized water. It was diluted 3 times. The equilibrium solubility study was ultimately executed. The water solubility profile of binary and ternary complexes was analyzed through spectrophotometry at 226 nm [24].

#### 2.2.5. Precompression Parameters

*(1) Micromeratic Properties*. *(a) Apparent Bulk Density*. The precompressed powder was weighed, and volume was calculated using a volumetric cylinder. The following formula was used to calculate bulk density: bulk density = mass of powder/volume of powder.

*(b) Tapped Density*. The precompressed and weighted powder was placed in a volumetric cylinder and tapped 100 times. Tapped density was calculated by the following formula: tapped density = mass of powder after tapping/volume of powder.

*(c) Compressibility Index*. The compressibility index (CI) was calculated using the following formula: CI = tapped density–bulk density/tapped density.

*(d) Hausner's Ratio*. Hausner's ratio was calculated by using the following formula: Hausner′s ratio = tapped density/bulk density

*(e) Angle of Repose*. The angle of repose is determined by fixed funnel methods, in which the sample is allowed to accumulate and form a conical heap on the surface. The height and radius of the heap were calculated. Values are put below the equation, and the angle of repose was determined. tan*θ* = *h*/*r*.

#### 2.2.6. Postcompression Parameters

*(1) Tablet Formation*. A direct compression method was adopted for preparing tablets of Glc and its complexes. In all these tablets, Glc, starch, PVP (polyvinyl pyrrolidine), lactose, and magnesium stearate were added as binders, disintegrating agents, and lubricating agents. These were homogenously mixed and sieved through a mesh of US 180 *μ*m. As shown in Table 1, each tablet weighed around 200 mg and contained around 10 mg of Glc [24].

*(2) Weight Variation, Hardness, and Friability*. For performing the weight variation test, 20 tablets were weighed individually and the average weight for each sample was calculated. Each tablet's weight was compared to the average weight. The hardness of the tablets was tested using a Monsanto hardness tester. The tablet was subjected to strength by placing between anvils, and the crushing force was recorded. Friability is the percentage loss of tablets' weight due to mechanical action. It was determined using a Roche Friabilator. For this purpose, 20 tablets were selected for each sample. After finding their initial weight, these were put in Roche Friabilator. Tablets were revolved for 4 min at 25 rpm. After the completion of the cycles, the tablets were cleaned with a brush to remove any powder or crumbs [25]. Percentage friability was calculated by applying the following formula:

(1) $$\begin{array}{c} \text{Percent friability}=\left(\frac{\text{loss in weight}}{\text{initial weight}}\right)\times 100. \end{array}$$

All values were expressed as mean ± standard error to the mean.

#### 2.2.7. *In Vitro* Drug Release Profile

In the *in vitro* drug release study on a pure drug, its binary and ternary complexes were undertaken using a paddle dissolution test apparatus. Each tablet sample was added to 900 mL of phosphate buffer solution (pH 7.4) as a dissolution medium at 37 ± 0.5°C. For making buffer, a 27.22 g of potassium dihydrogen phosphate solution was prepared in 1000 mL of deionized water. Similarly, a solution of 8 g sodium hydroxide was prepared in 1000 mL of deionized water. These solutions were mixed in 2000 mL of deionized water to make a 4 L buffer solution of pH 7.4. For carrying out the dissolution study of 20 tablets, 20 L buffer was prepared.

The stirring speed was maintained at 100 rpm. In all the experiments, 10 mL of the sample was withdrawn from each vessel at a time interval of 5, 15, 30, 60, 90, 120, 180, and 240 minutes and replaced by adding an equal volume of fresh buffer at each time to maintain the sink condition. The concentration of drug released from each tablet sample was probed with an ultraviolet spectrophotometer using a quartz cell of 0.2 cm. The absorbance was taken at 226 nm [26].

#### 2.2.8. Release Kinetic Analysis

*(1) Model-Dependent Approach*. To know about the drug release phenomenon, different mathematical models such as the zero-order model, first-order model, Higuchi model, Hixson Crowell model, and Korsmeyer–Pappas model were used [27].

*(a) First-Order Kinetics*.

(2) $$\begin{array}{c} l_{n}Q^{t}=l_{n}Q^{0}-K1^{t}. \end{array}$$

*(b) Zero-Order Kinetics*.

(3) $$\begin{array}{c} Q^{t}=Q^{0}-K^{0t}. \end{array}$$

*(c) Hixson Crowell Model*.

(4) $$\begin{array}{c} {M_{O}}^{1/3}-M^{1/3}=Kt. \end{array}$$

*(d) Higuchi Kinetic Model*.

(5) $$\begin{array}{c} Q^{t}={Kht}^{1-2}. \end{array}$$

*(e) Korsmeyer–Peppas Model*.

(6) $$\begin{array}{c} Qt=Ktn. \end{array}$$

Q = amount of drug discharge in time “*t*.” *K* = rate constant (kappa). *Q*^0^ = preliminary value of *Q*. *n* = release exponent.

*(2) Model-Independent Approach*. The model-independent approach requires similarity (*f*2) and difference factor (*f*1).

(7) $$\begin{array}{c} f2=50\times \log\left\{1+\left(\frac{1}{n}\right)St\right.=\ln{\left(R_{t}-T_{t}\right)}_{2}-0.5\times 100,\\ f1=\left\{\frac{\left[\sum_{t=1}^{n}\left|R_{t}-T_{t}\right.\right]}{\left[\sum_{t=1}^{n}R_{t}\right]}\right\}\times 100. \end{array}$$

#### 2.2.9. *In Vivo* Efficacy of Binary and Ternary Complexes of Glc

*(1) Animals*. Binary and ternary complexes of samples in different ratios (1 : 1, 1 : 9, 1 : 1 : 1, 1 : 3.6 : 3.6, and 1 : 9 : 1, respectively) prepared by PM and SE methods were selected for the investigation of antidiabetic activity in animal models (male rabbits weighing 1.0–1.5 kg). The rabbits were maintained under standard conditions in a cage system and acclimatized for a period of 7 days prior to the commencement of the experiment. The rabbits were fed with a standard pellet diet and water ad libitum and kept at room temperature in a cycle of 12 hours of dark and 12 hours of light. All rabbits were categorized into 11 groups having six animals in each. All the animals used in this study were obtained from the university's animal house. The permission for the animal study was obtained from the biochemistry department's ethics committee, faculty of science, Bahauddin Zakariya University, Multan, Pakistan, under letter no. 545/PEC/2019. The experiment was carried out in accordance with the U.K. Animals (Scientific Procedures) Act, 1986, and associated guidelines.

*(2) Diabetic Model*. The fasting blood glucose level of each rabbit was monitored using an Accu-Chek glucometer. Then, diabetes was induced in the rabbits using alloxan monohydrate (150 mg/kg) dissolved in 10 mL of a 5% freshly prepared saline solution [28]. To make the 5% saline solution, 3.3 g of NaCl was added into 150 mL of distilled water. A 3 mL solution of 100 mg alloxan per kg body weight was injected into the peritoneal cavity of each rabbit for 3 consecutive days. Before the injection of alloxan, the rabbits were fasted for 12 hrs. Each day, each rabbit was orally given a 2 mL solution of 500 mg glucose prepared in distilled water. After alloxan injection, the fasting blood glucose was systematically assessed at 1, 2, 3, 4, 6, 8, 12, 24, and 48 hrs through a glucometer by bolting a single drop of blood from the marginal vein of the rabbit ear [29]. The rabbits with glucose levels above 11.11 mmol/L were assumed as diabetic and included in our study.

*(3) Administration of Samples*. Diabetic rabbits were administered orally as solid pellets with10 mg/1.5 kg of pure Glc [30] with an equivalent amount of Glc-HP*β*CD-Tryp to groups I to XI. The rabbits were grouped as follows: group I: control group treated with 0.01 g (10 mg) of Glc; group II: rabbits treated with 0.02 g of the sample (PM 1 : 1); group III: rabbits treated with 0.02 g of the sample (SE 1 : 1); group IV: rabbits treated with 0.1 g of the sample (PM 1 : 9); group V: rabbits treated with 0.1 g of the sample (SE 1 : 9); group VI: rabbits treated with 0.03 g of the sample (PM 1 : 1 : 1); group VII: rabbits treated with 0.03 g of the sample (SE 1 : 1 : 1); group VIII: rabbits treated with 0.08 g of the sample (PM 1 : 3.6 : 3.6); group IX: rabbits treated with 0.08 g of the sample (SE 1 : 3.6 : 3.6); group X: rabbits treated with 0.11 g of the sample (PM 1 : 9 : 1); and group XI: rabbits treated with 0.11 g of the sample (SE 1 : 9 : 1).

Following administration, blood samples of the rabbits were collected from the marginal vein at 0, 1, 2, 3, 4, 6, 8, 12, and 24 hours. These blood samples were analyzed for glucose using the Accu-Chek glucometer. Mean and standard deviations were calculated. The following formula was used to calculate glucose reduction percentages:

(8) $$\begin{array}{c} \%-\text{age decrease in blood glucose level}\left(\text{BGL}\right)=\text{BGL at }t=0–\text{BGL at }\frac{t}{\text{BGL}}\text{ at }t=0. \end{array}$$

#### 2.2.10. Antioxidant Activity

Antioxidant activity was determined for diabetic rabbits (group 0), group I, group V, and group XII. Blood samples were taken from the central ear artery and stored in heparin-containing syringes. From these blood samples, plasma and erythrocyte lysates (ELs) were prepared. After removing plasma, packed erythrocytes were washed twice with 9 g/L NaCl solution and hemolyzed with cold distilled water. In these hemolysates, EL-catalase and EL-SOD activities were determined immediately [31].

EL-catalase activities were measured using a method developed by Beers and Sizer that is based upon the degradation of peroxide and was recorded spectrophotometrically at a wavelength of 240 nm. One unit of catalase is the amount of catalase that decomposes 1 *μ*mol H_2_O_2_/min under specific conditions [31].

EL-SOD activities were described following Beers and Sizer, in which the enzyme inhibits autooxidation of epinephrine and was recorded spectrophotometrically at 480 nm. One unit of SOD is defined as the amount of SOD that inhibits 50% of the autooxidation of epinephrine [31].

The plasma total antioxidant status (TAS) (mmol Trolox eq/L) was measured using the Erel spectrophotometric method [32], which is based on stable ABTS (2,2′-azino-bis (3-ethylbenzothiazoline-6-sulfonic acid) radical cations.

#### 2.2.11. Statistical Analysis

All the values of the current data are expressed as mean ± SD. The experiment results were assessed using the ANOVA (analysis of variance) single factor by SPSS-16. Furthermore, in order to compare which group of samples differs significantly, Tukey's multiple comparison test (for solubility and drug release test) was applied. Group comparisons in antioxidant activity were determined by the Mann–Whitney *U* test, followed by Student's *t*-test and Kruskal–Wallis test. Using Spearman's correlation test, correlation analysis was achieved.

## 3. Results and Discussion

### 3.1. Fourier Transform Infrared Spectroscopic Studies

Characteristic bands of pure Glc were observed at 3276.34 cm^−1^ corresponding to the –NH stretch of urea moiety, 1162.11 cm^−1^ due to symmetric stretching of S=O, and 1349.68 cm^−1^ due to asymmetric stretching of S=O. C=O stretching of carbonyl was observed between 1700 cm^−1^ and 1709 cm^−1^. –CH stretching peaks due to the aromatic ring of Glc were detected at 2935.87 cm^−1^ [33]. These were the major functional groups that were inclusively involved in complex formation. The FTIR spectrum of HP*β*CD indicated the –OH group in the wavenumber region of 3200 cm^−1^ to 3600 cm^−1^. C=O stretching vibrations were detected at 1031.63 cm^−1^. Our findings adequately showed the –CH peak at 2929.75 cm^−1^ [27]. The FTIR spectra of pure Tryp showed visible peaks at 1659.58 cm^−1^ due to –CO_2_ asymmetric stretch, 1453.66 cm^−1^ due to –CH mode of the indole ring, and 1582.10 cm^−1^ due to amide bond. Data showed that –NH stretching vibration was observed at 3400.71 cm^−1^ [34].

**Figure 2:**
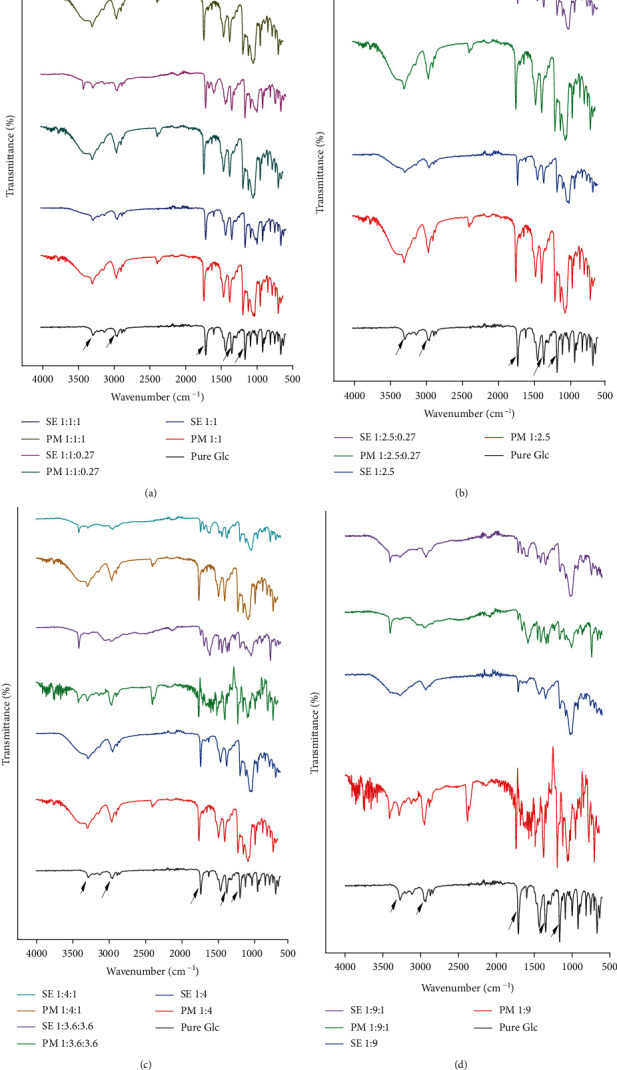
Comparison of FTIR spectra of pure Glc with (a) PM and SE 1 : 1, 1 : 1 : 1, and 1 : 1 : 0.27, (b) PM and SE 1 : 2.5 and 1 : 2.5 : 0.27, (c) PM and SE 1 : 4, 1 : 3.6 : 3.6, and 1 : 4 : 1, and (d) PM and SE 1 : 9 and 1 : 9 : 1.

ATR-FTIR spectra of Glc and its binary and ternary complexes with HP*β*CD and Tryp made by PM and SE are eminently presented in Figure 2. There is a slight shift in the carbonyl absorption band at 1709 cm^−1^and a significant reduction in intensity in all SE complexes as compared to PM complexes and pure Glc. The peaks due to the –NH group of Glc and –OH group of HP*β*CD at 3200 cm^−1^–3600 cm^−1^ were lost in the complexes of Glc and HP*β*CD in binary and ternary systems made by PM and SE. These showed bonding interactions among the –NH group of Glc and the –OH group of HP*β*CD, with altered frequencies of S=O, CO, and –CH stretching peaks of Glc. It should have appeared as a sharp shoulder in the spectrum of complexes if it was not altered by the complexation process. A completely different pattern of absorption bands was observed in the fingerprint region, including shifts of some bands, such as 1349.68 cm^−1^. The obtained IR spectra for Glc and HP*β*CD in binary and ternary complexes indicated slight modifications in comparison to those of free Glc and were generally consistent with previous reports on Glc and HP*β*CD complexes [35, 36]. Since the observed changes were almost identical to those reported in the literature for Glc and HP*β*CD complexes, it could be concluded that the Glc-HP*β*CD complex was formed.

The intensity of the band at 1453.66 cm^−1^ (−CH mode of indole ring) was observed to be decreased after complex formation in ternary complexes. Peak intensities due to the –NH stretch, –SO stretch, and –CH stretch of Glc and the CO of HP*β*CD were also found to be altered. The band at 1658 cm^−1^ representing the NH_3_^+^ asymmetric angular deformation disappeared in the inclusion complexes, and an intense band appeared at 1632 cm^−1^. This was related to the new chemical environment of amino acids, in which NH_3_^+^ formed a hydrogen bond with –OH groups of HP*β*CD. Our results are consistent with those of a previous study [37]. Similarly, the intensity of the amide bond of Tryp at 1582 cm^−1^ significantly decreased after complexation as compared to the physical mixture, with SE 1 : 9 : 1 yielding the best results. This decrease in respective intensities at 1582 cm^−1^ and 1453 cm^−1^ proved that tryptophan had successfully formed inclusion complexes with Glc and HP*β*CD. Thus, as evidenced by our findings, these changes support the complex formations between the drug and carriers.

### 3.2. X-Ray Diffractometry

**Figure 3:**
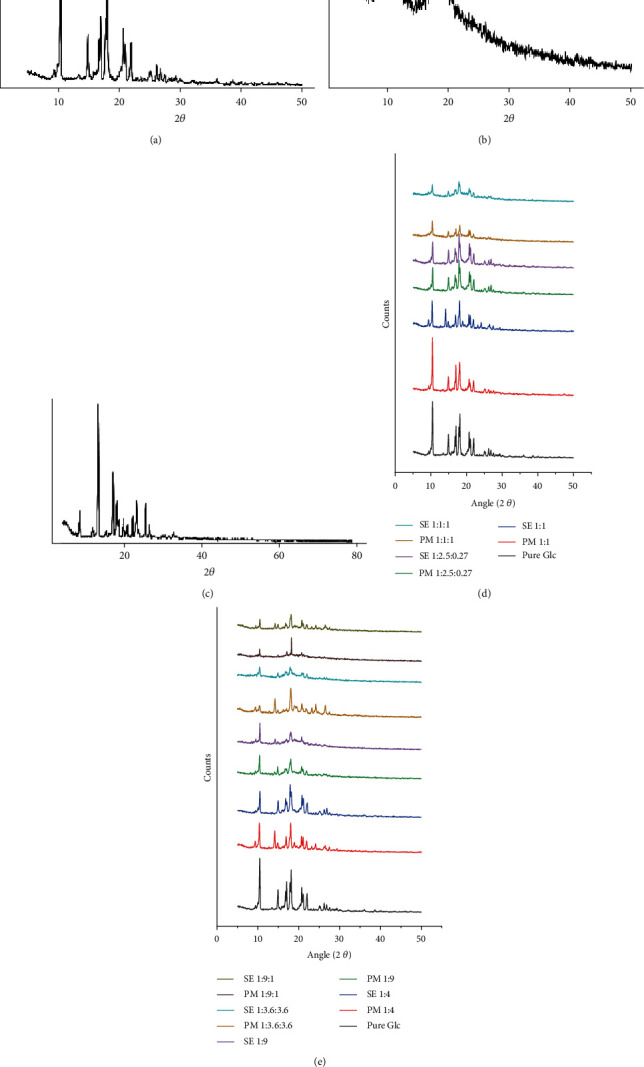
Demonstration of X-ray diffractograms of pure (a) Glc, (b) HP*β*CD, and (c) Tryp; XRD comparison graph of pure Glc with (d) 1 : 1 binary and 1 : 1 : 1 and 1 : 2.5 : 0.27 ternary systems and (e) 1 : 4, 1 : 9 binary and 1 : 3.6 : 3.6 and 1 : 9 : 1 ternary systems.

Seven samples of PM and SE were profoundly selected for XRD study, i.e., 1 : 1, 1 : 4, 1 : 9, 1 : 1 : 1, 1 : 2.5 : 0.27, 1 : 3.6 : 3.6, and 1 : 9 : 1.The X-ray diffraction peaks of pure Glc were observed at 2*θ* having intensity at 10.4° (2168), 14.9° (922), 17° (1245), 18° (1706), 20.7° (1024), 22° (762), and 26.2° (402). The highest peak was formed at 10.4°, which had an intensity of 2168. The Glc showed high-intensity peaks due to its crystalline nature (Figure 3(a)). HP*β*CD did not show any sharp peak in the diffractogram, which validates its amorphous nature (Figure 3(b)). X-ray diffractometry revealed high-intensity peaks at 9.4° (997), 14.2° (4337), 18° (2189), 19° (1285), 24° (1309), and 26° (1231). Thus, high-intensity peaks demonstrated the crystalline nature of Tryp (Figure 3(c)).

The comparison of powder X-ray diffraction patterns of Glc with its 1 : 1 binary and ternary complexes (1 : 2.5 : 0.27, 1 : 1 : 1) is shown (Figure 3(d)). Crystallinity can be determined by comparing some representative peak heights in the diffraction patterns of the binary and ternary systems with those of the reference. The crystalline nature of the complexes was less than that of the pure drug. The 1 : 1 : 1 ternary system showed the lowest values for intensity peaks, followed by the ternary system (1 : 2.5 : 0.27) and the 1 : 1 binary system. The decrease of intensity peaks was more pronounced in ternary complexes made by SE. The comparison of the powder X-ray diffraction patterns of Glc with its 1 : 4 and 1 : 9 binary and its 1 : 3.6 : 3.6 and 1 : 9 : 1 ternary complexes is shown (Figure 3(e)).

The 1 : 4 binary complexes made by physical mixture revealed the same intensity changes as SE 1 : 1. A decrease in intensity of some peaks with an increasing carrier ratio in binary complexes authenticated the amorphousness of the complexes. This showed an interaction between Glc and HP-*β*-CD polymer. Our results are comparable to Glc and *β* cyclodextrin complexes [38]. Crystallinity was much reduced in 1 : 9, which ratifies interactions between Glc and HP*β*CD in complex formation. An increase in intensity was observed more in 1 : 1 : 1 than in 1 : 9, which further validates the rise in the crystallinity nature. The least intensity was observed in 1 : 9 : 1. Thus, peak intensity decreases as the carrier's ratio increases. At 22° intensity, the peak in complexes was found to be totally diminished, which proved the interaction between the drug and both carriers.

Furthermore, the complexes with notably lower intensities had a lower number of signals, implying that the complexes were more amorphous than the drug. The extent of crystallinity influences the dissolution of the drug. An amorphous or metastable form will dissolve at the fastest rate because of its higher internal energy and greater molecular motion, which enhances the thermodynamic properties compared to crystalline materials [39].

Some changes in the peak position of Glc were adequately observed in PM and SE binary and ternary complexes. Pure Glc has prominent peaks at 10.4°, 14.9°, 17°, 18°, 20.7°, 22°, etc. They were clearly spotted at the same position in PM and SE, but the intensities were observed to be significantly decreased. In complexes made by SE, the intensity of Glc peaks was significantly reduced. We can deduce from the previous observations that the drug's crystalline nature was preserved. However, the relative reduction of the diffraction intensity of Glc in complexes indicates that the quality of the crystals was reduced. Some new diffraction peaks were also observed in complexes made by SE, which proves the formation of inclusion complexes. The appearance of new diffraction peaks in the spectrum of the “interacted mixture,” as well as the shift of the guest molecules' characteristic peaks and changes in their relative intensity, all indicate the formation of a new solid phase and support the actual inclusion complex formation [40]. The results of this study revealed that Glc is present in a partially crystalline or microcrystalline form in the PM and SE binary and ternary complexes. Our findings are consistent with previous research on Glc complexes with polyethylene glycol (PEG 6000) [41].

### 3.3. Scanning Electron Microscopic Studies

**Figure 4:**
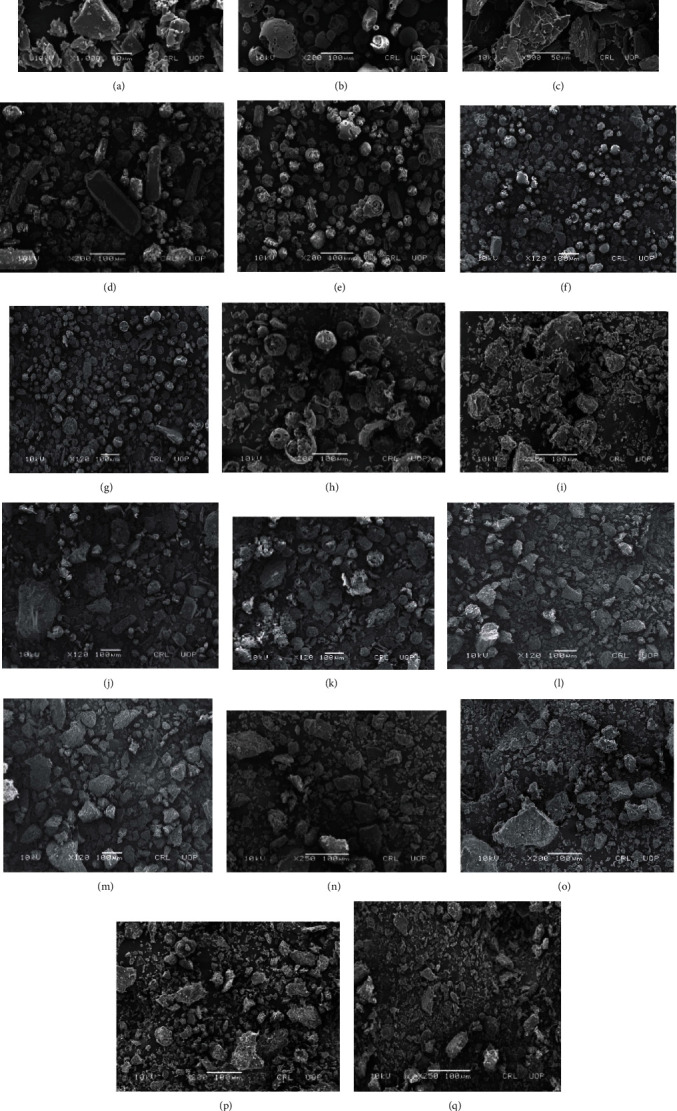
SEM photomicrographs of (a) pure Glc, (b) HP*β*CD, (c) Tryp, (d) PM 1 : 1, (e) SE 1 : 1, (f) PM 1 : 4, (g) SE 1 : 4, (h) PM 1 : 9, (i) SE 1 : 9, (j) PM 1 : 1 : 1, (k) SE 1 : 1 : 1, (l) PM 1 : 2.5 : 0.27, (m) SE 1 : 2.5 : 0.27, (n) PM 1 : 3.6 : 3.6, (o) SE 1 : 3.6 : 3.6, (p) PM 1 : 9 : 1, and (q) SE 1 : 9 : 1.

Scanning electron photomicrograph studies were executed on four samples, i.e., 1 : 1, 1 : 9, 1 : 3.6 : 3.6, and 1 : 9 : 1, made by PM and SE. SEM analysis of pure components and their complexes were taken at ×200 100 *μ*m, ×250 100 *μ*m, ×500 50 *μ*m, ×1000 10 *μ*m, ×1500 10 *μ*m, and ×2500 10 *μ*m. Glc was found to possess irregularly shaped crystals of different sizes. Lamellar-shaped crystals were also observed at this resolution. Small crystals were found to stick to large, irregular, and lamellar-shaped crystals (Figure 4(a)). Scanning electron photomicrographs of pure HP*β*CD revealed globular crystals (Figure 4(b)). Tryp exhibited small thin crystals of flaky structures (Figure 4(c)). SEM images of binary complexes of Glc and HP*β*CD in 1 : 1, 1 : 4, and 1 : 9 made by PM and SE showed lamellar and globular-shaped crystals. Small irregular-shaped particles appeared to be embedded in the interior of large globular crystals. Thus, it was determined that Glc and HP*β*CD might have some interaction. SEM images of PM and SE in 1 : 9 revealed reduced crystallinity compared to 1 : 1 and 1 : 4, respectively (Figure 4).

SEM images of ternary complexes of Glc, HP-*β*-CD, and Tryp (1 : 1 : 1, 1 : 2.5 : 0.27, 1 : 3.6 : 3.6, and 1 : 9 : 1) made by PM and SE showed random crystals of lamellar, globular, thin, and flaky structures. With the increasing ratio of carriers in complexes, the crystalline nature of components was presumed to be reduced. Agglomeration was clearly visible at high resolution. Thus, it evidenced the complexation of pure Glc with both carriers. SEM images of PM and SE of 1 : 9 : 1 demonstrated much-reduced crystallinity than 1 : 3.6 : 3.6 and other binary complexes. Increased carrier ratios in complexes lead to categorically reduced crystallinity (Figure 4). Research has shown that a modification in the shape of the drug particles was suggestive of a new solid state. Hence, the changes in the particle shape and size as observed in the SEM scans of the binary and ternary systems suggested the change in the physical properties of the drug and carrier, implying the formation of drug-carrier complexes [42]. According to a recent study, HP*β*CD provided a beadless and uniform structure by forming inclusion complex nanofibers, which contributed to improving water solubility and dissolution tests [43].

### 3.4. Equilibrium Solubility Studies

Cyclodextrin is regarded as potentially able to amend the pharmacokinetic characteristics of a drug by forming specific inclusion complexes. The hydrophobic central cavity of cyclodextrin trapped hydrophobic active groups of drugs, whereas its hydrophilic groups were exposed to the external environment. Consequently, the cyclodextrin-drug complex entity is set up. Because of the unique structural properties of various cyclodextrin derivatives, chemotherapeutic drug solubility can be increased. For example, docetaxel (a poorly soluble drug) can be made more soluble by forming inclusion complexes with HP*β*CD, *β*CD, and sulfobutyl*β*CD [44].

**Figure 5:**
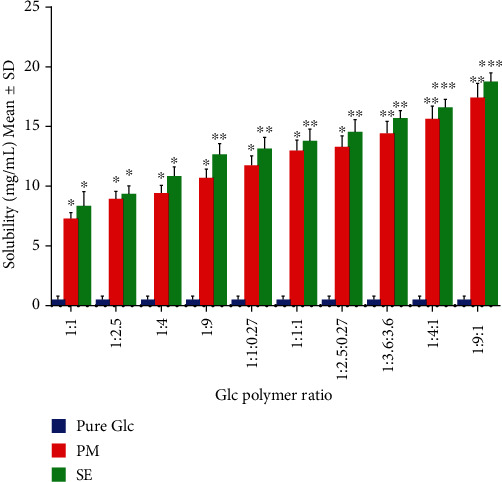
Equilibrium solubility of Glc in binary and ternary complexes with HP*β*CD and Tryp. ^∗^*p* < 0.05, ^∗∗^*p* < 0.01, and ^∗∗∗^*p* < 0.001 represent statistical significance.

The Glc solubility was equitably recorded in 0.5 mg/mL of deionized water at 37°C. According to graphical representation (Figure 5), complexes stipulated significantly higher solubility than pure Glc (*p* < 0.05). Likewise, ternary complexes made by the SE method demonstrated higher solubility (*p* < 0.001) than binary complexes (*p* < 0.05) as compared to pure Glc. The concentration of the drug released from binary complexes of physical mixtures was observed between 7.31 ± 0.50 to 10.7 ± 0.60 mg/mL, and that of SE was between 8.36 ± 1.20 and 12.69 ± 0.60 mg/mL.

The concentration of drug released from ternary complexes made by physical mixtures was discerned at 11.75 ± 0.90–17.4 ± 1.00 mg/mL, while that of SE was found to be 13.2 ± 0.90 to 18.73 ± 1.00 mg/mL. Ternary complexes were more soluble (16.9–18.23 times in 1 : 9 : 1) than binary complexes (10.2–12.9 times in 1 : 9). The solubility was determined in the descending order of SE > PM > pure Glc, which was substantially all inclusive. Of all the preparations, 1 : 9 : 1 showed the best solubility profile. When compared to pure Glc, the concentration of drugs released from PM and SE 1 : 9 : 1 was 17.40 ± 1.0 and 18.73 ± 1.0, respectively. Recent studies found that HP*β*CD and *β*CD increased the solubility of difenoconazole in water [45, 46]. In the solvent evaporation method, a drug is dispersed in a water-soluble carrier by a suitable means. Reduction in particle size to submicron or molecular dimension improves the dissolution rate. Another justification is the conversion of the drug from an amorphous to a crystalline form, which has higher solubility and wettability via a hydrophilic carrier, as observed in an earlier research study [45].

PM and SE mixtures of all samples showed a substantial increase in equilibrium solubility as the carrier ratio increased. The increase in solubility was due to the amorphous nature of the prepared samples or the inhibition of crystallization. Generally, the solubility profile of binary and ternary complexes prepared by PM and SE methods agreed with the data of XRD, FTIR, and SEM, which indicated that both PM and SE improved the solubility profile of Glc.

### 3.5. Characterization of Tablets

#### 3.5.1. Precompression Parameters

*(1) Micromeritics of the Powder Sample*. The powder mixture of all batches showed good flow characteristics (Table 2). Bulk density varied from 0.423 ± 0.34 to 0.592 ± 0.23, tapped density from 0.534 ± 0.45 to 0.593 ± 0.24, Carr's index ranges between 12.34 ± 0.34 and 17.33 ± 0.46, Hausner's ratio from 1.132 ± 0.34 to 1.198 ± 0.34, and angle of repose 19.34 ± 0.34 to 24.53 ± 0.75.

#### 3.5.2. Postcompression Parameters

*(1) Weight Variation, Hardness, and Percentage Friability of Tablets*. For each sample, 20 tablets were prepared and evaluated assiduously for weight variation, hardness, and friability. For this purpose, their average and standard deviation were determined. All the tablets had a weight variation of 199.2 ± 1.06 mg to 199.74 ± 0.90 mg. The hardness of tablets was observed at 2.8 ± 0.68 to 4.27 ± 0.81 kg/cm^2^. The tablets showed less than 1% friability, which manifests good mechanical characteristics (Table 3).

### 3.6. *In Vitro* Drug Release Profile

The binary and ternary complexes of Glc with HP*β*CD and Tryp at different drug-carrier ratios exhibited an eloquently maximum drug release compared to pure Glc. The drug release from PM samples was found to be substantially higher with an increased carriers' ratio compared to pure Glc. Thus, ternary complexes adequately showed a higher drug release profile, i.e., 65.4 ± 1.00%, compared to binary complexes (34.7 ± 0.98%).

**Figure 6:**
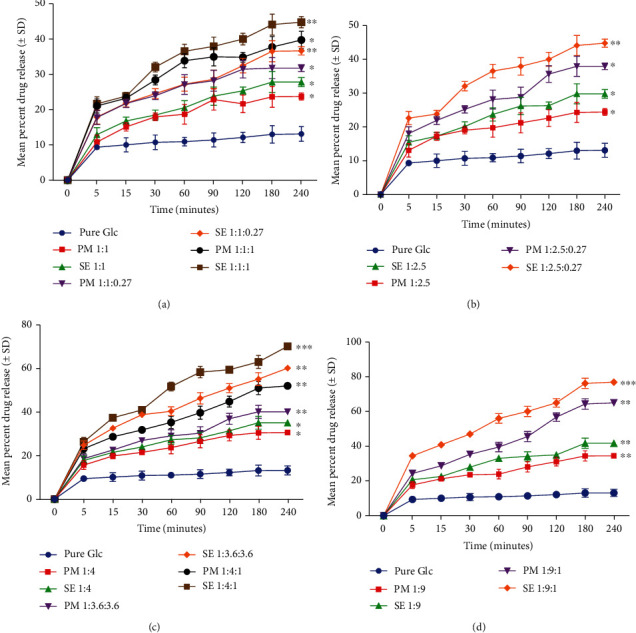
Comparison of the drug release profile of Glc, HP*β*CD, and Tryp prepared by PM and SE methods: (a) 1 : 1 binary, 1 : 1 : 0.27, and 1 : 1 : 1 ternary systems, (b) 1 : 2.5 binary and 1 : 2.5 : 0.27 ternary systems, (c) 1 : 4 binary, 1 : 3.6 : 3.6, and 1 : 4 : 1 ternary systems, (d) 1 : 9 binary and 1 : 9 : 1 ternary systems. ^∗^*p* < 0.05, ^∗∗^*p* < 0.01, and ^∗∗∗^*p* < 0.001 denote statistical significance.

Binary and ternary complexes formed by SE demonstrated maximum drug release (36 ± 1.0%–77.4 ± 0.05%) compared to PM. Thus, ternary complexes made by the SE method demonstrated a significantly higher drug release profile than PM mixtures when compared to pure Glc. A graphical comparison between pure Glc and binary and ternary systems in different polymer ratios is shown (Figure 6). The SE of Glc with HP*β*CD and Tryp exhibited considerably enhanced drug release compared to pure Glc and PM. From all samples, 1 : 9 : 1 showed a maximum drug release percentage. When compared to pure Glc, it showed 65.4 ± 1.0 and 77.40 ± 1.0 mean percent drug release. All preparations showed a substantial increase in the percent drug release (Figure 6), which could be attributed to the amorphous nature of complexes or the inhibition of crystallization provided by carriers [47], as opposed to Glc, which is completely crystalline.

Research studies have reported that ternary complexes of the model drug naproxen-proline with either arginine or tryptophan have increased solubility and dissolution profile [21]. In comparison to pure crystalline drugs, complexes of indomethacin and carbamazepine with tryptophan, arginine, phenylalanine, and tyrosine demonstrated significantly higher solubility and dissolution profiles [48]. In addition, recent research studies have shown that HP*β*CD in the form of inclusion complexes with difenoconazole and thiophanate methyl demonstrated a faster dissolution than pure drugs [45, 49]. The drug release results were fitted into kinetic models (zero, first, Higuchi, Korsmeyer-Peppas, and Hixon-Crowel) (Table 4), and based on the value of the regression coefficient (*R*^2^), it was determined that the majority of the formulations follows the Higuchi and Korsmeyer-Peppas models. All findings were evaluated using ANOVA (one way), which showed that there is a significant difference between formulations (*p* ≤ 0.043).

### 3.7. *In Vivo* Biological Activity of Gliclazide and Its Binary and Ternary Complexes

#### 3.7.1. Induction of Diabetes

Before induction of diabetes, the fasting blood glucose level of each rabbit was estimated. The blood sugar levels ranged from 4.8 mmol/L to 6.4 mmol/L. After 1^st^ hour following Alloxan, the values did not vary significantly from the fasting values. The values at the 2^nd^ and 3^rd^ hours were significantly higher (*p* ≤ 0.05), followed by a decline at the 4^th^ hour (5.4 ± 0.11 mmol/L to 7.8 ± 0.16 mmol/L) and 6^th^ hour (5.24 ± 0.17 mmol/L to 8.7 ± 0.11 mmol/L) when compared to the 2^nd^ hour (5.97 ± 0.12 mmol/L to 7.62 ± 0.17 mmol/L), but were then significantly higher at the 8^th^ hour (5.23 ± 0.11 mmol/L to 9.35 ± 0.33 mg/dL). All these values were significantly higher (*p* ≤ 0.05) when compared to the initial fasting blood glucose level. From the 8^th^ hour posttreatment, the blood glucose level increased significantly up to 48 hours (*p* ≤ 0.05). Thus, animal models with blood glucose levels > 11.11 mmol/L were presumed to be diabetic (Supplementary Figure 1).

#### 3.7.2. Antidiabetic Activity

**Figure 7:**
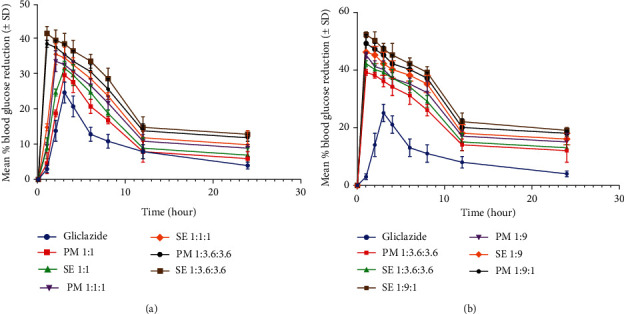
Comparison of antidiabetic activity of pure Glc with (a) 1 : 1 binary, 1 : 1 : 1, and 1 : 3.6 : 3.6 ternary complexes and (b) 1 : 9 binary, 1 : 3.6 : 3.6, and 1 : 9 : 1 ternary complexes prepared by the PM and SE methods. 1 : 9 : 1 ternary complexes showed the highest blood glucose reduction in the first hour (*p* < 0.001) as compared to the free drug (*p* > 0.05).

The antidiabetic activity of Glc and its binary and ternary complexes was probed in animal models of albino rabbits. For this purpose, five samples were made by PM and SE were scrutinized for antidiabetic evaluation. Mean percentage reductions in blood glucose levels were calculated and are represented graphically in Figure 7. After 3 hrs, group I (the control group) had a peak level of blood glucose reduction 1.38 ± 0.16% (8.7 mmol/L). PM and SE 1 : 1 of groups II and III showed a maximum mean percentage glucose reduction of 1.6 ± 0.11% and 1.77 ± 0.11% (78 mmol/L and 7.5 mmol/L) after 3 hrs, respectively. When compared to those of group I, these ratios indicated that a maximum glucose reduction was possible. Groups VI and VII demonstrated higher blood glucose reduction peaks at 1.8 ± 0.15% and 2 ± 0.16% (7.4 mmol/L and 7.38 mmol/L) after 2 hrs, respectively. After 1 hr, groups VIII and IX signaled maximum peak reductions for blood glucose levels of 2.16 ± 0.05% and 2.3 ± 0.12% (6.6 mmol/L and 6.5 mmol/L), respectively. Thus, PM and SE of 1 : 3.6 : 3.6 for groups VIIII and IX promised a smaller percentage glucose reduction compared to binary and ternary complexes of 1 : 9 and 1 : 9 : 1, respectively. PM of 1 : 9 and SE 1 : 9 in groups IV and V showed maximum blood glucose reduction of 2.5 ± 0.06% and 2.55 ± 0.04% (6.16 mmol/L and 6.05 mmol/L) after 1 h, respectively. PM of 1 : 9 : 1 and SE 1 : 9 : 1 in groups X and XI were found to show the highest blood glucose reduction at 2.72 ± 0.16% and 2.8 ± 0.05% (5.7 mmol/L and 5.3 mmol/L) after 1 h, respectively. The mean maximum percentage reduction in the blood glucose level of ternary complexes made by PM and SE showed a highly significant difference than that of pure drugs. Hence, the binary and ternary systems of Glc alter its antihyperglycemic activity as well as the duration of action (Figure 7). Thus, it is hypothesized that the percentage decrease in the blood glucose level for binary and ternary complexes is higher compared to pure drugs.

Ternary complexes denoted maximum blood glucose reduction compared to binary complexes and pure drugs. With respect to the maximum percentage decrease in the blood glucose level, it is evident in Figure 7 that ternary complexes had the lowest value for the time required to reduce the blood glucose level, followed by binary complexes and then the free drug. Recent research studies reported that cyclodextrins improve the biological activity of drugs in the form of inclusion complexes, such as complexes of *β*-cyclodextrin and cayazine which displayed better herbicidal activity as compared to pure cayazine [50]. Similarly, HP*β*CD improved the antimicrobial activity of anacardic acid by forming inclusion complexes [19].

### 3.8. Antioxidant Activity

**Figure 8:**
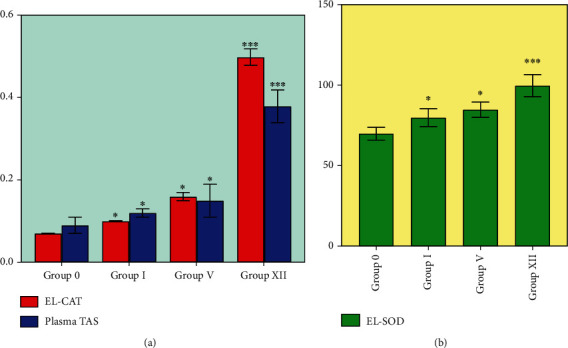
Antioxidant parameters in plasma and erythrocyte containing lysates of the study groups (mean ± SD). (a) CAT activity in erythrocytes and plasma TAS levels. (b) SOD activity in erythrocytes. Expressions are U·mL^−1^ for EL-SOD, U·mg^−1^ protein for EL-CAT, and mmol Trolox for TAS. ^∗^*p* < 0.05 and ^∗∗∗^*p* < 0.001 denote statistical significance.

Diabetic rabbits (group 0) revealed a marked decrease in plasma TAS, EL-CAT, and EL-SOD levels, which were restored by Glc alone (*p* ≤ 0.05) and in the form of complexes. Glc (group I) treatment alone significantly increases the level of TAS, EL-CAT, and EL-SOD. However, when combined with HP*β*CD in a binary system (group V), these antioxidant markers increased compared to those of group I. HP*β*CD acts as a secondary antioxidant. It augmented the antioxidant activity of Glc in diabetic rabbits. Group XII (ternary complex) achieved the best results with a *p* value less than 0.001 (Figure 8) because Tryp in the ternary system remarkably increased the level of antioxidant markers as compared to other treated groups. Alper et al. investigated the possible effect of the selegiline and monoamine oxidase inhibitor in combination with Glc on oxidative stress prevention in diabetic albino rats. They found that while this combined treatment contributes to improving antioxidant status, the EL-CAT activity in diabetic rats was not significantly higher as compared to the control level. These findings are in contradiction with our findings [51]. The complexation of HP*β*CD and Tryp with Glc increases the levels of EL-CAT, EL-SOD, and TAS.

The increased formation of reactive oxygen species (ROS) occurs under diabetes conditions, affecting antioxidant status. This oxidative damage may play a vital role in the development of major macro- and microvascular diseases like atherosclerosis. Lipid peroxide levels increase in diabetic patients' circulatory systems. Acute glucose levels have been linked to lipid peroxidation, and recent investigations have shown that this glucose load reduces the human antioxidant defense mechanism. Glc inhibits LDL oxidation *in vitro* [6]. The marked increase in antioxidant status observed in diabetic rabbits treated with Glc alone and complexes is consistent with the findings of Mourya et al. [52], who observed increased antioxidant parameters such as TAS, malondialdehyde (MDA), SOD, and thiols in diabetic patients following Glc administration.

A previous study showed that HP*β*CD acts as a secondary antioxidant, increasing the system's natural antioxidant capacity. Furthermore, this study found that substrates have antioxidant properties embedded in the hydrophobic moiety of cyclodextrin, resulting in the nonavailability of these natural antioxidants to the oxidative enzymes [53].

Hoseini et al. investigated the effects of dietary Tryp on the oxidative condition of the fish's intestinal mucosa. They concluded that consuming this amino acid at the recommended dietary amount could improve the antioxidant status in animal models [54].

Glc, HP*β*CD, and Tryp possess antioxidant activity. The combination of Glc therapy with both carriers in the form of complexes contributes to the control of oxidative stress and demonstrated better outcomes in improving antioxidant parameters in diabetic patients. This combination of the drug with both carriers may improve diabetic patients' survival rates and has implications for further clinical trials.

## 4. Conclusions

To conclude, the value of this work has been to discuss the methods of preparation, solubility, drug release profile, bioactivity, and antioxidant activity of Glc, HP-*β*-CD, and Tryp complexes, as well as to compare the efficiency of binary and ternary complexes. Furthermore, our research can be viewed as a type of screening in which we selected the best ratio and method for achieving the par excellence results. The binary and ternary complexes of Glc with HP*β*CD and Tryp were prepared using the PM and SE methods. The possibility to use the SE method enables more accurate product definitions.

Ternary complexes prepared using the SE method showed a higher solubility than pure Glc and other binary complexes. The *in vitro* drug release profile can be improved as the carrier ratio increases in complexes. The FTIR results showed that adequate complex formation occurred between Glc and carriers. According to X-ray diffraction and SEM image studies, increasing the carrier ratio causes the crystalline nature of Glc to change to amorphousness, substantiating the complexation phenomenon between pure Glc and carriers. Additionally, an antioxidant study revealed that Glc, along with both carriers, has a high potential as a free radical scavenger and may improve TAS, SOD, and CAT levels, which are reduced in diabetics. This could substantiate complexes' secondary benefit. The *in vivo* antidiabetic activity of pure Glc and the complexes indicates that Glc's binary and ternary systems may improve antihyperglycemic activity while shortening its duration of action. Our findings indicate that increasing the ratios of Tryp and HP*β*CD in a constant amount of drug may restore and improve Glc's carrier-binding and medicinal properties. Our findings showed that PM and SE 1 : 9 : 1 performed well in all preparations when compared to the other binary and ternary complexes. Thus, cyclodextrins and amino acids have the potential to serve as the foundation for a new platform technique to overcome the challenges associated with poorly soluble drugs. Our findings warrant future studies of Glc solubility with other amino acids, as well as additional animal studies and clinical trials.

## Figures and Tables

**Table 1:** Composition of the tablet.

Samples	Calculation of active (mg)	Lactose (mg)	PVP (mg)	Starch (mg)	Magnesium stearate (mg)
Control	10	68	56	56	10
1 : 1	10 + 10 = 20	60	55	55	10
1 : 2.5	10 + 25 = 35	55	50	50	10
1 : 4	10 + 40 = 50	50	45	45	10
1 : 9	10 + 90 = 100	40	25	25	10
1 : 1 : 0.27	10 + 10 + 2.7 = 22.7	60	50	50	10
1 : 1 : 1	10 + 10 + 10 = 30	57.3	55	55	10
1 : 2.5 : 0.27	10 + 25 + 2.7 = 37.7	52.3	50	50	10
1 : 3.6 : 3.6	10 + 36 + 36 = 82	38	35	35	10
1 : 4 : 1	10 + 40 + 10 = 60	50	40	40	10
1 : 9 : 1	10 + 90 + 10 = 110	30	25	25	10

**Table 2:** Micromeritic properties of powder.

Batch code	Bulk density	Tapped density	Cars index	Hausner ratio	Angle of repose
Control	0.423 ± 0.34	0.535 ± 0.34	12.34 ± 0.34	1.132 ± 0.34	19.34 ± 0.34
1 : 1	0.532 ± 0.34	0.588 ± 0.23	14.24 ± 0.34	1.134 ± 0.56	23.45 ± 0.67
1 : 2.5	0.478 ± 0.66	0.539 ± 0.23	15.35 ± 0.24	1.134 ± 0.54	22.45 ± 0.74
1 : 4	0.590 ± 0.45	0.593 ± 0.24	15.45 ± 0.68	1.198 ± 0.34	24.2 ± 0.83
1 : 9	0.455 ± 0.46	0.534 ± 0.45	16.35 ± 0.46	1.145 ± 0.35	20.34 ± 0.35
1 : 1 : 0.27	0.582 ± 0.65	0.55 ± 0.35	17.33 ± 0.46	1.155 ± 0.24	21.45 ± 0.34
1 : 1 : 1	0.498 ± 0.63	0.535 ± 0.35	14.46 ± 0.35	1.185 ± 0.35	22.45 ± 0.68
1 : 2.5 : 0.27	0.533 ± 0.34	0.545 ± 0.53	16.35 ± 0.64	1.157 ± 0.35	24.53 ± 0.75
1 : 3.6 : 3.6	0.496 ± 0.34	0.535 ± 0.56	15.35 ± 0.56	1.175 ± 0.35	23.53 ± 0.45
1 : 4 : 1	0.592 ± 0.23	0.553 ± 0.34	14.56 ± 0.46	1.167 ± 0.45	20.55 ± 0.67
1 : 9 : 1	0.545 ± 0.34	0.535 ± 0.754	14.53 ± 0.56	1.156 ± 0.355	22.43 ± 0.46

**Table 3:** Hardness and percent friability of tablets prepared in different ratio.

Samples	Weight variation (mg)	Hardness (kg/cm^2^)	Percent friability
Control (gliclazide)	199.56 ± 0.73	4.27 ± 0.81	0.41 ± 0.21
PM 1 : 1	199.68 ± 0.72	4.09 ± 0.50	0.51 ± 0.17
PM 1 : 2.5	199.55 ± 0.85	3.26 ± 0.72	0.56 ± 0.16
PM 1 : 4	199.44 ± 0.75	3.29 ± 0.89	0.44 ± 0.25
PM 1 : 9	199.21 ± 0.98	3.09 ± 0.89	0.45 ± 0.13
PM 1 : 1 : 0.27	199.14 ± 1.05	3.34 ± 0.84	0.45 ± 0.13
PM 1 : 1 : 1	199.57 ± 0.84	2.8 ± 0.68	0.48 ± 0.24
PM 1 : 2.5 : 0.27	199.46 ± 0.75	3.44 ± 0.98	0.44 ± 0.18
PM 1 : 3.6 : 3.6	199.57 ± 0.84	2.99 ± 0.74	0.53 ± 0.28
PM 1 : 4 : 1	199.61 ± 0.90	2.93 ± 0.85	0.53 ± 0.25
PM 1 : 9 : 1	199.59 ± 0.73	3.15 ± 0.60	0.46 ± 0.25
SE 1 : 1	199.74 ± 0.90	4.12 ± 0.91	0.48 ± 0.20
SE 1 : 2.5	199.44 ± 0.94	4.03 ± 0.72	0.38 ± 0.21
SE 1 : 4	199.2 ± 1.06	3.5 ± 0.22	0.44 ± 0.20
SE 1 : 9	199.14 ± 1.00	2.96 ± 0.53	0.46 ± 0.16
SE 1 : 1 : 0.27	199.18 ± 0.87	3.25 ± 0.54	0.50 ± 0.17
SE 1 : 1 : 1	199.13 ± 1.25	3.6 ± 0.8	0.51 ± 0.22
SE 1 : 2.5 : 0.27	199.21 ± 1.24	3.4 ± 0.38	0.48 ± 0.20
SE 1 : 3.6 : 3.6	199.06 ± 1.31	2.92 ± 0.71	0.55 ± 0.14
SE 1 : 4 : 1	199.09 ± 1.16	2.86 ± 0.66	0.45 ± 0.23
SE 1 : 9 : 1	199.09 ± 1.18	2.88 ± 0.51	0.50 ± 0.16

PM: prepared by physical mixing method; SE: prepared by solvent evaporation method.

**Table 4:** Kinetic models of drug release.

Formulation code	Zero-order model		First-order model		Higuchi model		Korsmeyer-Peppas model			Hixson-Crowell model	
	*R* ^2^	*K*_0_	*R* ^2^	*K*_1_	*R* ^2^	*k*_H_	*R* ^2^	*k*_KP_	*n*	*R* ^2^	*k*_HC_
Control (gli)	0.89	3.93	0.99	0.54	0.99	12.20	0.93	4.87	0.52	0.88	0.01
PM 1 : 1	0.98	2.24	0.94	0.65	0.98	15.35	0.94	5.58	0.60	0.95	0.01
PM 1 : 2.5	0.78	4.87	0.85	0.18	0.94	14.66	0.94	4.84	0.44	0.84	0.05
PM 1 : 4	0.88	5.58	0.86	0.62	0.95	14.09	0.95	2.89	0.38	0.92	0.07
PM 1 : 9	0.92	4.84	0.85	0.679	0.97	23.32	0.94	3.27	0.36	0.91	4.87
PM 1 : 1 : 0.27	0.96	2.89	0.86	0.05	0.98	2.20	0.96	14.03	0.45	0.80	5.58
PM 1 : 1 : 1	0.98	3.27	0.84	0.03	0.96	3.78	0.95	15.66	0.45	0.83	4.84
PM 1 : 2.5 : 0.27	0.83	5.48	0.96	0.46	0.95	4.61	0.93	15.71	0.48	0.87	2.89
PM 1 : 3.6 : 3.6	0.89	6.93	0.96	0.54	0.96	3.20	0.96	11.49	0.54	0.88	0.54
PM 1 : 4 : 1	0.98	7.24	0.93	0.55	0.98	3.35	0.94	10.11	0.55	0.95	0.55
PM 1 : 9 : 1	0.78	8.87	0.86	0.48	0.93	13.66	0.93	28.64	0.48	0.84	0.48
SE 1 : 1	0.88	4.58	0.87	0.62	0.93	18.09	0.95	38.95	0.62	0.92	0.62
SE 1 : 2.5	0.92	8.84	0.83	0.49	0.92	19.32	0.94	42.29	0.36	0.91	0.54
SE 1 : 4	0.86	6.89	0.84	0.44	0.99	12.20	0.95	14.03	0.45	0.80	0.01
SE 1 : 9	0.98	4.27	0.88	0.56	0.99	13.78	0.94	15.66	0.45	0.83	0.02
SE 1 : 1 : 0.27	0.93	4.48	0.95	0.66	0.94	14.61	0.94	15.71	0.48	0.87	0.02
SE 1 : 1 : 1	0.99	5.93	0.97	0.54	0.97	2.20	0.93	11.49	0.52	0.88	0.01
SE 1 : 2.5 : 0.27	0.98	5.24	0.94	0.55	0.97	13.35	0.95	10.11	0.60	0.95	0.01
SE 1 : 3.6 : 3.6	0.98	6.87	0.87	0.58	0.98	24.66	0.95	28.64	0.44	0.84	0.05
SE 1 : 4 : 1	0.98	5.58	0.86	0.32	0.95	28.09	0.93	38.95	0.38	0.92	0.07
SE 1 : 9:1	0.92	7.84	0.84	0.59	0.94	29.32	0.94	42.29	0.36	0.91	0.07

## Data Availability

Datasets generated during and/or analyzed for this study project have been included in the main text. Data pertaining to ethics and, or any other supplementary materials, data are available from the corresponding authors upon reasonable request.

## Supplementary Materials

Supplementary Materials Supplementary file includes data about induction of diabetes in rabbits using Alloxan. Graphical representation revealed that all values at different time zones (1^st^, 2^nd^, 3^rd^, 4^th^, 5^th^, 6^th^, 7^th^, and 8^th^ hours) were significantly higher compared to initial fasting blood glucose levels (Supplementary Figure 1).
