# Supplementary material for: Gliclazide in Binary and Ternary Systems Improves Physicochemical Properties, Bioactivity, and Antioxidant Activity
Source: Oxid Med Cell Longev. 2022 Nov 25;2022:2100092. doi: 10.1155/2022/2100092 (PMC9718633; doi:10.1155/2022/2100092)
Supplement: Supplementary Materials — Supplementary file includes data about induction of diabetes in rabbits using Alloxan. Graphical representation revealed that all values at different time zones (1st, 2nd, 3rd, 4th, 5th, 6th, 7th, and 8th hours) were significantly higher compared to initial fasting blood glucose levels (Supplementary Figure 1). [file 2100092.f1.docx]

**Gliclazide in Binary and Ternary Systems Improves Physicochemical Properties, Bioactivity, and Antioxidant Activity**

**Muhammad Ibrahim^1^, Shehla Munir^1^, Sarfraz Ahmed^2^, Adeel Hussain Chughtai^3,^ Waqas Ahmad^4^, Jallat Khan^5^, Mogana Das Murtey^6^, Hira Ijaz^7^, Suvash Chandra Ojha^8,9*^**

^1^Department of Biochemistry, Bahauddin Zakariya University, Multan, 60800, Pakistan

^2^Department of Basic Sciences, University of Veterinary and Animal Sciences, Narowal, 51600, Narowal, Pakistan

^3^Institute of Chemical Sciences, Bahauddin Zakariya University, Multan, 60800, Pakistan

^4^Department of Clinical Sciences, University of Veterinary and Animal Sciences, Narowal, 51600, Narowal, Pakistan

^5^Department of Chemistry, Khwaja Fareed University of Engineering & Information Technology, 64200, Rahim Yar Khan, Pakistan

^6^Basic Sciences and Oral Biology Unit, School of Dental Sciences, Health Campus, Universiti Sains Malaysia, 16150, Kubang Kerian, Kelantan Malaysia

^7^Department of Pharmacy, University of Faisalabad, Faisalabad, Pakistan

^8^Department of Infectious Diseases, The Affiliated Hospital of Southwest Medical University, Luzhou 646000, China

^9^Southwest Medical University, Jiangyang District, Luzhou 646000, Sichuan, China

***Correspondence:**

Suvash Chandra Ojha

[suvash_ojha@swmu.edu.cn](mailto:suvash_ojha@swmu.edu.cn)

**Figure 1:** Trend of immediate changes in blood glucose level of 11 groups of albino rabbits following administration of Alloxan monohydrate (150 mg/Kg body weight). P ≤ 0.05 was statistically significant.
